# Internal Herniation and Small Bowel Strangulation Following Sacrocervicopexy With Mesh: A Case Report and Review of the Literature

**DOI:** 10.7759/cureus.105435

**Published:** 2026-03-18

**Authors:** Kristin M Knickerbocker, Shane Skibba, Nicholas K Lago, Mario Gomez

**Affiliations:** 1 General Surgery Residency, Broward Health Medical Center, Fort Lauderdale, USA; 2 General Surgery, Florida Atlantic University Charles E. Schmidt College of Medicine, Boca Raton, USA; 3 Surgery, Florida International University Herbert Wertheim College of Medicine, Miami, USA

**Keywords:** closed-loop obstruction, exploratory laparotomy, small bowel surgery, strangulated hernia, vaginal prolapse repair

## Abstract

This case report presents evidence supporting further investigation into the surgical steps and complications of sacrocervicopexy (SCerP).

The patient is a 72-year-old woman who presented to the emergency department (ED) with sudden-onset severe abdominal pain, 15 months after undergoing SCerP for vaginal prolapse. She was taken to the operating room for the reduction of a retroperitoneal herniation and subsequently required small bowel resection due to ischemic bowel.

There is a scarcity of documented cases of retroperitoneal herniation following SCerP, resulting in a lack of established guidelines for management of the retroperitoneal defect created during the index operation.

Retroperitoneal herniation after SCerP is rarely reported, and the role of retroperitoneal closure following SCerP remains unclear. Further research is needed to assess outcomes following SCerP without retroperitoneal closure.

## Introduction

Internal hernias, defined as protrusions of a viscus through a peritoneal or mesenteric defect within the abdominal or pelvic cavity, are a very rare cause of small bowel obstruction [1]. Previous research has identified internal hernias in 0.6%-5.8% of small bowel obstructions [1,2], with an overall incidence estimated between 0.2% and 0.9% [1]. Internal hernias can be categorized by location, including paraduodenal, pericecal, foramen of Winslow, transmesenteric, transmesocolic, intersigmoid, and retroanastomotic hernias [1,3]. Although retroperitoneal herniation is not thoroughly described in the literature, a case of small bowel herniation through a retroperitoneal defect following surgery involving retroperitoneal dissection has been reported [4]. Patients presenting with clinical signs of small bowel obstruction can be evaluated using CT imaging with intravenous contrast.

On CT imaging, signs concerning for small bowel obstruction due to a retroperitoneal hernia are similar to findings in other small bowel obstructions, including distended loops of bowel, a transition point between dilated and decompressed loops, crowded loops within a possible herniated sac, and abnormalities of the mesenteric vessels such as engorgement or twisting [1,5].

Abdominal sacrocolpopexy (SCP) was first described in 1957. Since then, it has become the gold standard procedure for apical vaginal prolapse and is often performed minimally invasively using laparoscopy or robotic-assisted laparoscopy [6]. SCP is performed by suturing mesh between the anterior longitudinal ligament of the sacrum and the fibromuscular layer of the anterior and posterior vaginal walls [7]. A very similar procedure, sacrocervicopexy (SCerP), involves attachment of the uterine cervix, rather than the vaginal wall, to the anterior longitudinal ligament of the sacrum. Both procedures require dissection into the retroperitoneal space over the sacral promontory, with direct attachment of a mesh graft to the anterior longitudinal ligament. The retroperitoneum is sometimes closed over the exposed graft material; however, the literature notes a lack of evidence regarding the necessity of retroperitoneal closure [7,8]. One study found no significant difference in outcomes with or without reperitonization of the mesh [9]. Nevertheless, some surgeons may elect to reapproximate the retroperitoneum due to the potential for bowel obstruction, a rare complication of SCP and SCerP [7,10]. Our case describes a scenario of small bowel obstruction due to an internal hernia in a patient with a history of pelvic organ prolapse treated with laparoscopic SCerP.

## Case presentation

**Figure 1:**
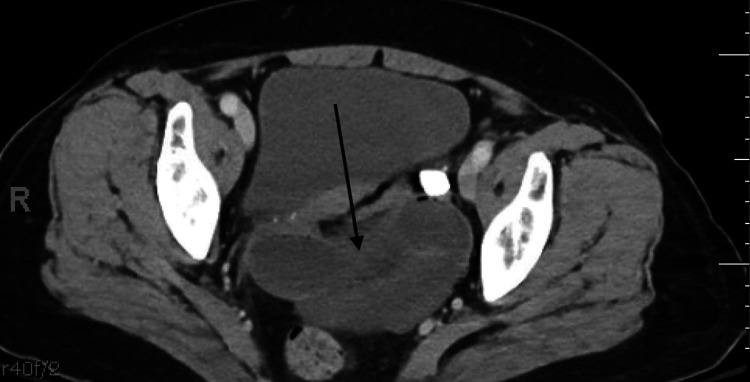
Abdominal and pelvic CT with intravenous contrast from initial presentation (transverse view)

**Figure 2:**
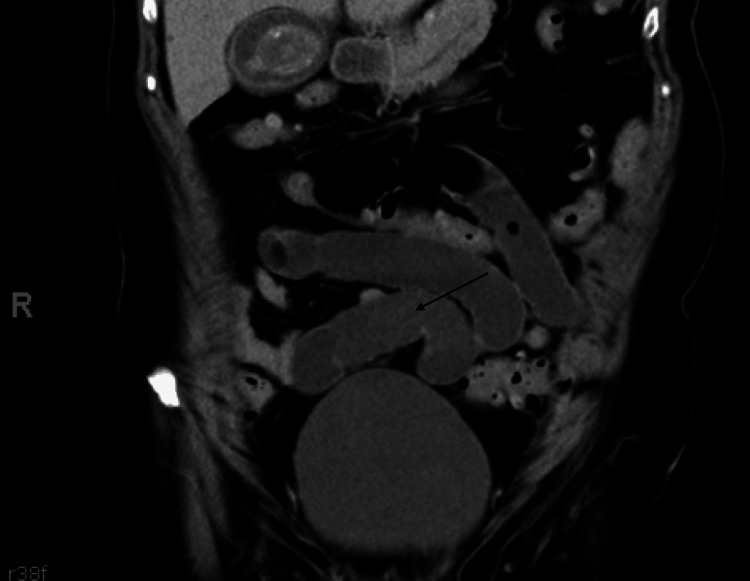
Abdominal and pelvic CT with intravenous contrast from initial presentation (coronal view)

The patient is a 72-year-old woman with a history of ulcerative colitis and pelvic organ prolapse treated with laparoscopic SCerP, presenting to the emergency department (ED) with approximately 12 hours of severe bilateral lower abdominal pain, associated with nausea and vomiting. She remained hemodynamically stable, afebrile, and with normal oxygen saturation on room air throughout her ED course. The patient had not passed flatus or had a bowel movement for an estimated 24 hours. On physical examination, she had significant tenderness in the right lower quadrant without signs of peritonitis, with some focal guarding in the same region. The abdomen was soft but moderately distended. Workup included complete blood count (CBC), complete metabolic panel (CMP), lactate level, urinalysis, chest X-ray, and CT imaging of the abdomen and pelvis with intravenous contrast. Laboratory studies revealed no leukocytosis; hemoglobin, liver function tests, and urinalysis were all within normal limits. Lactic acid level was also normal (Table 1). CT imaging demonstrated a distended, fluid-filled small bowel with a small amount of left-sided pelvic free fluid (Figure 1 and Figure 2, arrows). A loop of bowel in the posterior lower abdomen appeared decompressed on either side. At this stage, the differential diagnosis included internal hernia, bowel perforation, and small bowel obstruction. Given these findings, the patient was taken to the operating room for exploratory laparotomy.

**Table 1:** Lab values on arrival

Lab test	Value	Reference values
Lactic acid (serum)	2.1	0.5-2.2 mmol/L
White blood cell count	8,500	4,000-11,000 cells/µL
Hemoglobin	13	12.0-15.5 g/dL
Leukocyte esterase (urine)	Negative	Negative
Nitrite (urine)	Negative	Negative
Lipase	10	0-160 units/L
Aspartate aminotransferase	39	8-33 units/L
Alanine aminotransferase	24	7-55 units/L
Alkaline phosphatase	61	44-147 international units/L
Blood urea nitrogen	22	7-20 mg/dL
Creatinine	0.6	0.6-1.1 mg/dL

**Figure 3:**
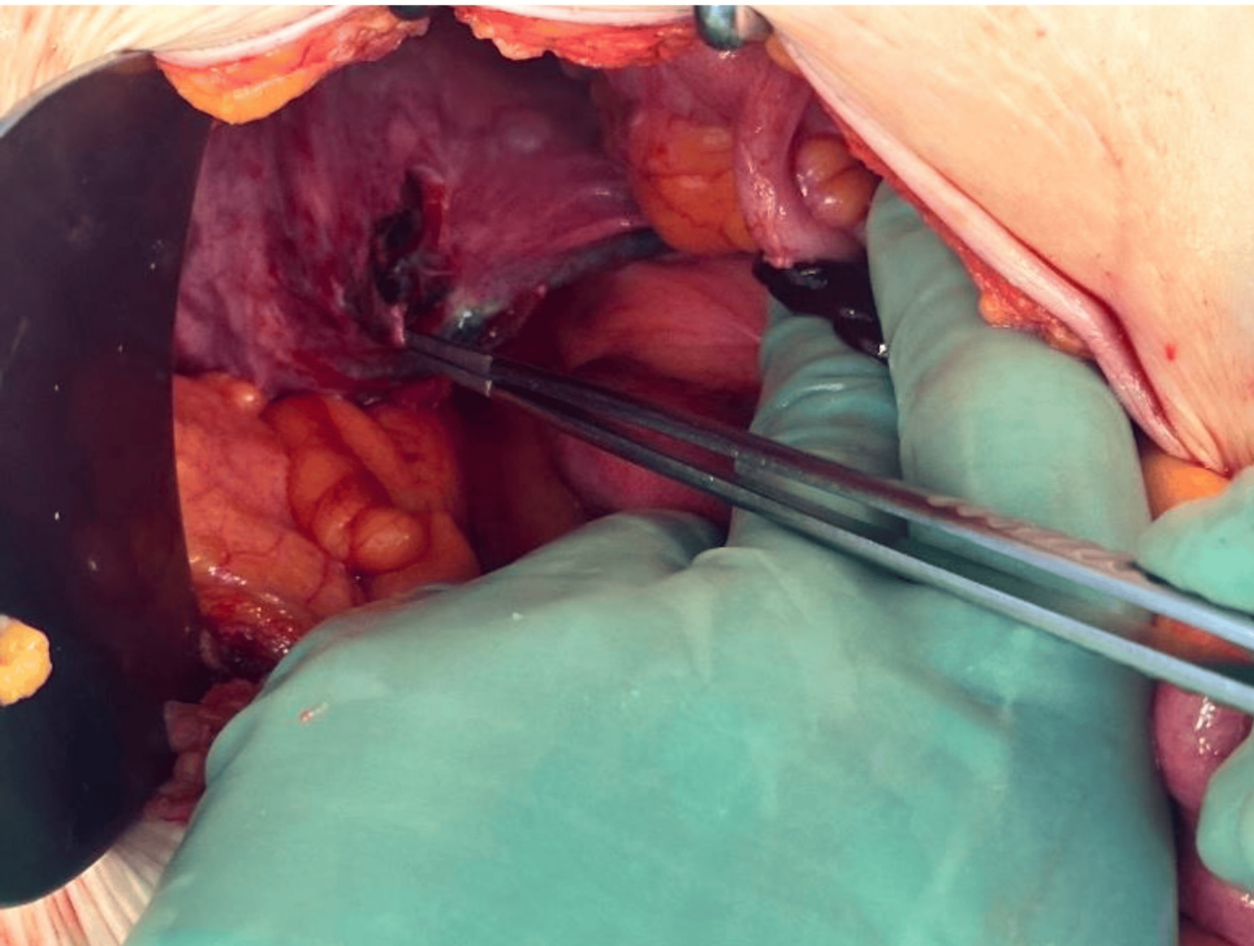
Intraoperative peritoneal defect associated with SCerP SCerP: sacrocervicopexy.

**Figure 4:**
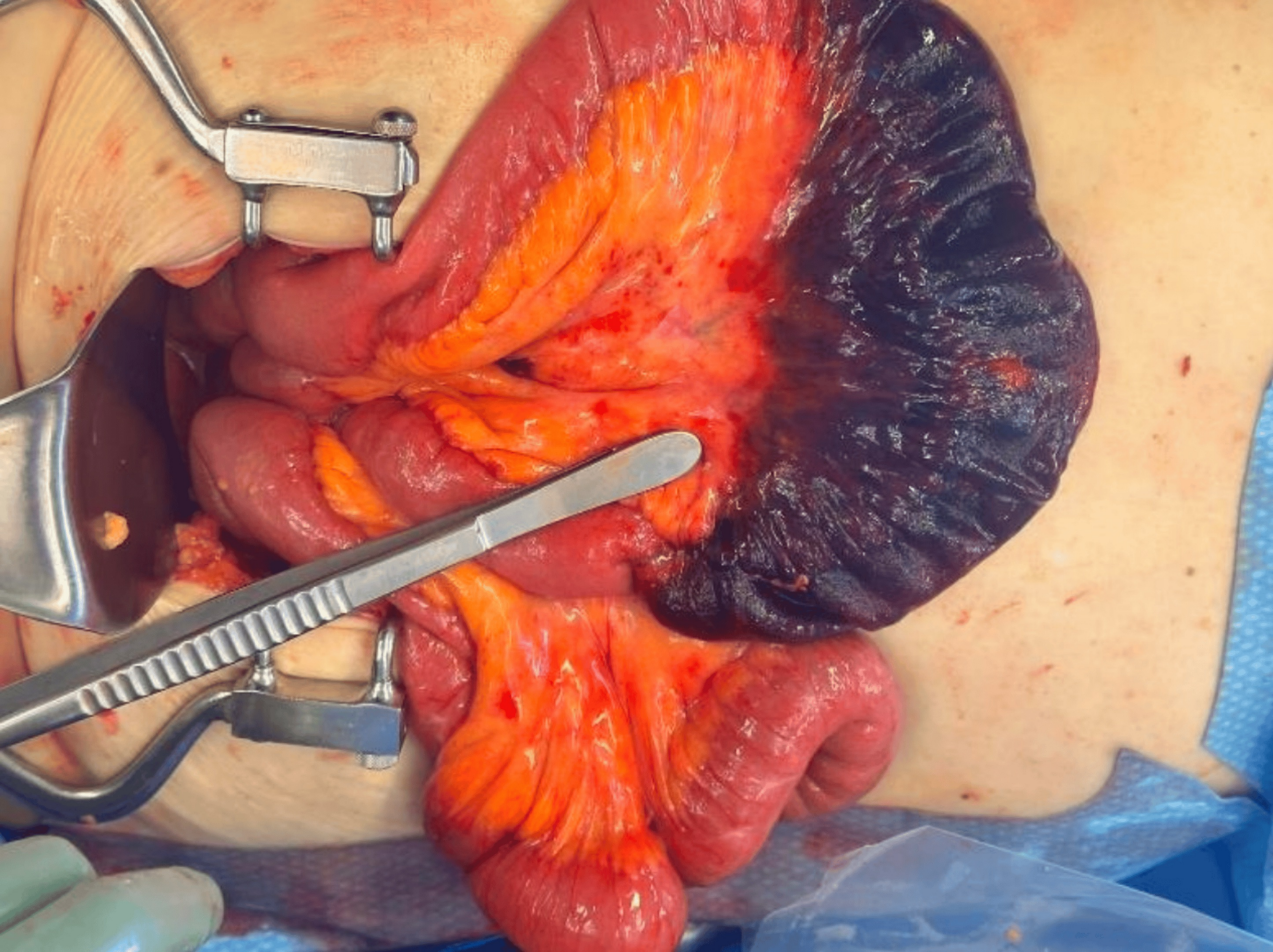
Intraoperative herniated small bowel after reduction of retroperitoneal hernia reduction

In the operating room, a low midline periumbilical incision was made, and the small bowel was examined from the ligament of Treitz distally. Adhesions were noted around the lower posterior abdominal wall near the SCerP mesh repair. A defect of approximately 3 cm was identified in the peritoneum at the site of the mesh, through which about 20 cm of small bowel had herniated and become strangulated (Figure 3). The herniated bowel was reduced and noted to be frankly ischemic (Figure 4). Approximately 20 cm of the small bowel was resected, and a side-to-side anastomosis was performed using gastrointestinal anastomosis and thoracoabdominal staplers. The peritoneal defect associated with the SCerP mesh was closed using a running 0 polydioxanone suture. The abdomen was irrigated with normal saline and closed in a standard multilayer fashion. The patient was transferred to the post-anesthesia care unit, extubated, and remained hemodynamically stable. The immediate postoperative course was uneventful.

Postoperatively, the patient remained NPO with a nasogastric tube in place while awaiting return of bowel function. She was ambulating by postoperative day 2 and was cleared by the physical therapy team on postoperative day 3. The nasogastric tube was removed on postoperative day 3 after a four-hour clamp trial, once the patient began passing flatus. Her diet was advanced to clear liquids on postoperative day 4. The patient’s abdomen remained soft with appropriate tenderness throughout her hospital stay. Laboratory studies, including CBC and CMP, remained stable. By postoperative day 6, the patient was tolerating a regular diet, passing flatus, and having non-bloody bowel movements. She was deemed stable for discharge with appropriate follow-up on postoperative day 6.

## Discussion

This case presents a very rare cause of small bowel obstruction due to internal herniation following laparoscopic SCerP. Although internal hernias account for approximately 5.8% of small bowel obstructions [2], retroperitoneal internal herniation is rarely described in the literature. Our review identified several cases of retroperitoneal herniation [11-13]; however, these were primarily associated with renal transplant surgery or traumatic injury involving the retroperitoneal space. Only a few cases of internal herniation resulting in small bowel obstruction following SCP or SCerP have been reported [4,14].

The mechanism underlying this case may relate to the dissection of the retroperitoneum over the sacral promontory during SCerP. Closure of the retroperitoneum during SCP or SCerP remains controversial [7]. Many surgeons elect to close the retroperitoneum over the mesh due to concerns about potential small bowel obstruction [10]. Previous studies addressing this issue have not consistently demonstrated an increased risk of obstruction in the absence of reperitonization of the mesh [9,15].

This case underscores the need for further investigation into the surgical reperitonization of mesh in SCP and SCerP. Although the risk of retroperitoneal herniation following SCerP is exceedingly rare, the potential consequences can be severe. Furthermore, procedures such as SCP and SCerP are being performed more frequently, as they have become the gold standard for treating pelvic organ prolapse [8,11]. Further research with larger study populations is needed to determine the true risk of retroperitoneal herniation following SCP or SCerP and to help reduce morbidity and mortality. While larger case series have examined rates of small bowel obstruction after SCP, for example, a series of 3,231 patients with a maximum follow-up of 10 years, these studies do not specify whether operative interventions were performed for obstructions specifically caused by internal herniation [16]. Future studies should carefully assess the mechanism of bowel obstruction to accurately investigate the incidence of internal herniation in this population.

## Conclusions

There is a lack of sufficient data in the literature regarding herniation through retroperitoneal defects left after SCP or SCerP. Although rare, this case highlights the importance of addressing potential complications and may help guide surgical steps during SCerP. Currently, retroperitoneal closure during SCP and SCerP is left to the surgeon’s discretion. Because few cases of internal retroperitoneal herniation following SCerP have been documented, there are no established standards for retroperitoneal closure or for revision at the time of hernia reduction. Based on this case, we recommend closing the retroperitoneum during the index operation to reduce the potential space for internal herniation, although large-scale studies are not yet available to support this recommendation. Further investigation into the specific mechanisms of bowel obstruction is needed to better define outcomes and postoperative management for patients undergoing SCP or SCerP without retroperitoneal closure. Multidisciplinary collaboration between surgeons, radiologists, and gastroenterologists may help decrease the risk of internal herniation and improve patient outcomes.
